# Effect of vitamin D supplementation on glycemic control in patients with type 2 diabetes: a systematic review of interventional studies

**DOI:** 10.1186/s40200-015-0130-9

**Published:** 2015-02-12

**Authors:** Nisha Nigil Haroon, Ammepa Anton, Jisha John, Madhukar Mittal

**Affiliations:** Division of Endocrinology, Department of Medicine, University of Toronto, Toronto, ON Canada; Toronto Western Hospital, University Health Network, Toronto, ON Canada; Department of Medicine, Wayne State University, Detroit, MI USA; Endocrinology Unit, Department of Medicine, King George Medical University, Lucknow, India

**Keywords:** Cholecalciferol, Vitamin D, Glycemic control, Insulin sensitivity, Type 2 diabetes

## Abstract

**Background:**

Diabetes and vitamin D deficiency are global epidemics. Researchers have long been exploring the role of potentially modifiable factors to manage type 2 diabetes. We conducted a systematic review of prospective studies and randomized controlled trials that involved vitamin D supplementation and specifically intended to study glycemic outcomes related to type 2 diabetes.

**Methods:**

Two authors independently searched Medline and PubMed for longitudinal studies that had assessed the effect of vitamin D supplements on glycemic control, insulin resistance and beta-cell dysfunction in patients with diabetes.

**Results:**

Seventeen randomized control trials and seven longitudinal studies with a minimum follow-up of one month were included.

Results of the various short-term studies (follow up ≤ 3 months) suggested that vitamin D supplementation had a positive impact on glycemic control and metabolic parameters such as insulin resistance and beta cell dysfunction. However, the evidence was weak due to the low methodological quality of the studies. There was no significant effect on HbA1c, beta cell function and insulin resistance in the long-term studies (follow up > 3 months). There existed heterogeneity in the methodology of the studies, inclusion criteria, mode of supplementation of vitamin D and the duration of follow up.

**Conclusions:**

Current evidence based on randomized controlled trials and longitudinal studies do not support the notion that vitamin D supplementation can improve hyperglycemia, beta cell secretion or insulin sensitivity in patients with type 2 diabetes. Large-scale trials with proper study design, optimal vitamin D supplementation and longer follow up need to be conducted.

## Background

Diabetes is now widely prevalent globally [1]. Presently, around 285 million people have diabetes and this number is expected to reach 438 million by the year 2030 [2]. More alarmingly, many people are developing type 2 diabetes early in their lives. Achieving excellent glycemic control is crucial in the management of diabetes as well as preventing the onset of serious and life threatening complications of diabetes [3,4]. Despite the advances in the diagnosis and management of diabetes, achieving normoglycemia or optimal glycemic control is still considered challenging [5]. This is because care of type 2 diabetes warrants intense life-style adaptations, polypharmacy and insulin centered regimens. Conventional oral anti-diabetic medications are associated with hypoglycemias. Besides, insulin treatment has been linked to poor compliance, weight gain and possibly adverse cardiovascular outcomes. In addition, progressive beta-cell dysfunction and insulin resistance can make anti-diabetic agents less effective [6]. Despite large-scale educational campaigns and behavioral interventions, treatment adherence is only around 60% [7]. Moreover, newer anti-diabetic drugs such as incretin analogs and ultra short acting insulin analogs are expensive and hence many patients in the developing world where type 2 diabetes is prevalent cannot afford these medications. Besides, the long-term safety of the newer agents is still being explored. Also, no complete cure has yet been discovered for 2 diabetes.

Given the existence of many such challenges in the management if diabetes, researchers have been exploring the role of modifiable factors to manage type 2 diabetes. Vitamin D insufficiency and deficiency are being increasingly recognized world-wide [8]. Serum 25 (OH) D levels have even been linked to mortality in the general population [9]. Vitamin D level in plasma has been linked to the occurrence of metabolic syndrome and insulin resistance [10]. Though epidemiological studies demonstrate an association between low serum 25(OH) vitamin D and glucose intolerance, intervention trials using vitamin D have produced mixed results [11,12]. Epidemiological data also suggest a possible link between low vitamin D and diabetic complications such as nephropathy, neuropathy and retinopathy [11].

Recent observational data reports a beneficial effect of vitamin D on preventing the onset of diabetes [13]. But the potential benefits of vitamin D supplementation on glycemic control are still debated. A meta-analysis in 2012 that included longitudinal studies and randomized controlled trials (RCTs) reported a small improvement on fasting glucose and insulin resistance but no beneficial effect was seen on HbA1c [14]. However, studies included in this meta-analysis were heterogeneous in terms of the study subjects as healthy subjects, and those with impaired fasting glucose or type 2 diabetes were included. Clearly long-term studies were lacking. Further, the dose of supplemental vitamin D and duration of follow-up varied widely across the studies. The number of eligible studies was also small and data on HbA1C, a better marker of glycemic status was available only from four studies. Moreover, many studies did not analyze the effect of all possible confounders. In addition, more studies have been published in the two years since this review [11,15-23]. So it is important to update available evidence in this regard.

### Aims

Thus we conducted a systematic review of prospective studies and randomized controlled trials that assessed the role of vitamin D supplementation on glycemic outcomes related to type 2 diabetes. The purpose of our review is to synopsize the present knowledge on this topic.

## Materials and methods

### Selection criteria

#### Inclusion criteria

We considered studies that assessed the effect of vitamin D supplementation on glycemic control with a minimum follow-up period of one month as eligible. Studies had to have more than seventy percent of subjects with type 2 diabetes and be published as a full-text original article. The search was restricted to human studies published in English language. No restrictions were applied regarding geography or gender of the subjects. Eligible studies had to have reported at least one of the following primary outcomes of interest: insulin sensitivity and insulin secretion by homeostasis model assessment (HOMA-IR (insulin resistance) or B (beta-cell function)) and/or hemoglobin A1c (HbA1c).

#### Exclusion criteria

Review articles, studies done in children and adolescents, studies with lack of longitudinal follow-up, case reports, editorials and studies with follow-up less than 1 month were excluded. We also excluded studies that did not include any therapeutic intervention with vitamin D and studies in which subjects had gestational diabetes, post partum diabetes, diabetic nephropathy, type 1 diabetes and prediabetes. Studies that assessed incident diabetes as the primary outcome and studies in which patients had no diabetes were also excluded. If there were more than one publication from the same group and had similar interventions, the study with the maximum duration of follow-up and number of subjects was included [22].

We used the Preferred Reporting Items for Systematic Reviews and Meta-analyses statement to guide the reporting of this systematic review [24].

### Search strategy

**Figure 1:**
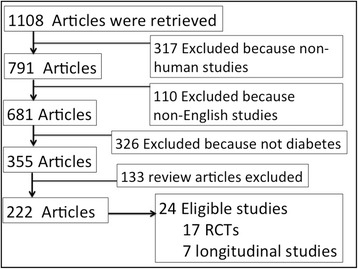
**Flow chart for identifying eligible studies.**

Two authors conducted the literature search independently. Systematic searches of MEDLINE and EMBASE were conducted from inception to July 31, 2014. The main aim was to identify randomized controlled trials that assessed the effect of vitamin D supplementation on glycemic control in patients with type 2 diabetes. The search strategy is outlined in Figure 1.

The search strategy used diabetes and vitamin D related search terms with glycemic control related search terms using a Boolean operator “AND”. The keywords and Medical Subject Headings were: ((“vitamin D” OR “cholecalciferol” OR “vitamin D3” OR “vitamin D2” OR “calcitriol” OR “ergocalciferol” OR “one-alpha-hydroxycholecalciferol” OR “doxercalciferol” OR “alphacalcidol” OR “alfacalcidol”) AND (“diabetes” OR “T2DM” OR “hyperglycemia” OR “hyperglycaemia” OR “dysglycemia” OR “diabetes mellitus” OR “diabetic”) AND (“glycemic control” OR “glycemic” OR “HbA1c” OR “control” OR “insulin sensitivity” OR “beta cell function” OR “euglycemia” OR “insulin resistance” OR “insulin” OR “HOMA” OR “fasting glucose” OR “FBS” OR “post prandial” OR “insulin secretion” OR “interventional” OR “insulin sensitivity” OR “glucose” OR “glucose homeostasis” OR “glycaemic control” OR “glucose tolerance” OR “effects” OR “changes”)) NOT ((“pregnancy” OR “gestational” OR “gestational diabetes”) OR (“associations” OR “association”) OR (“type-1 diabetes” OR “T1DM” OR “type 1 diabetes” OR “diabetes type 1” OR “children” OR “adolescents”) OR (“pre diabetes” OR “pre-diabetes” OR “prediabetes”) OR (“meta-analysis”) OR (“polycystic”) OR (“healthy” OR “nondiabetic”)).

We screened the reference lists of the eligible primary studies, narrative and systematic reviews so as to identify other candidate studies. Two authors independently assessed all retrieved records on titles and abstracts. Disagreements were resolved by discussion. If required, a third independent author was consulted to reach consensus. We aligned our final choice on the assessment of the full-text articles by two independent authors. A study was included if the earlier described inclusion criteria were fulfilled. Two authors independently extracted data from the selected studies. Data on study characteristics such as the number of patients, study design, country of origin, mean age, and use of vitamin D, and were collected whenever available. Information on baseline HbA1c, 25-OH-vitamin D levels, and homeostasis model assessment index of beta cell function (HOMA-B) and insulin resistance (HOMA-IR) and longitudinal change in all these parameters were also extracted whenever possible. HOMA-B and HOMA-IR are able to provide estimates of steady state beta cell function and insulin sensitivity and these are derived as percentages of a normal reference population. Studies with more than 3 months of follow-up were considered as long-term studies and those with a follow-up of three months or less were considered as short-term studies.

### Quality assessment

We used the Newcastle–Ottawa Scale to address the quality of the eligible studies and the Jadad scale to assess the quality of the RCTs [25,26]. The Newcastle–Ottawa Scale employs a star scoring system to judge the study aspects including comparability, selection of groups, and assessment of the outcome of interest. The quality of the definition of selection groups is graded from 0–4, comparability from 0–2 and ascertainment of exposures and outcomes from 0–3. Higher points indicate that the study has good quality. Two authors conducted the quality assessment independently and disagreements were discussed until a consensus was reached.

## Results

The search strategy is shown in Figure 1. Studies with more than 3 months of follow-up were considered as long-term studies and those with a follow-up of three months or less were considered as short-term studies. Sixteen studies were short-term studies. The remaining studies were long –term studies with follow up ranging from sixteen weeks to 18 months. There were two publications from the same research group based in Iran and hence the study with the maximum duration of follow-up, compete data and maximum number of subjects were included [22]. We identified twenty-four studies including 17 RCTs and 7 longitudinal studies as eligible based on the pre-defined inclusion –exclusion criteria [11,15-23,27-41]. Half of the studies were (13/24) conducted in Caucasians.

### Clinical and biochemical characteristics of the short-term studies

There were 16 short term-studies and 13 amongst them were randomized controlled trials. Data are shown in Table 1. The sample size ranged from 10–119. One study had included only women [27]. Most studies used oral cholecalciferol as supplemental vitamin D. The dose of cholecalciferol ranged from 400 IU to 5600 daily or 60, 000 IU weekly. Calcitriol, the active form of vitamin D was used in three studies [18,37]. Vitamin D2 was used in one study [33]. A single dose intramuscular injection of vitamin D3 was used in three studies [21,28,29]. None of the studies had used doxercalciferol or alphacalcidol.

**Table 1:** Clinical and biochemical characteristics of the short-term studies**

**Author, year, country**	**Study design, N**	**Duration**	**Intervention**	**Age (years)**	**Change in HbA1c and HOMA**	**Comments**
Parekh [28], 2010, India	RCT, N = 28	4 weeks	300000 IU vitamin D IM	No data	HbA1c 7.58 ± 0.57 vs. 7.67 ± 0.61, p=.393. No difference in HOMA-IR	No improvement in HbA1c and HOMA-IR
Borissova [27], 2003, Bulgaria	Longitudinal study, N = 10	1 month	Vitamin D3 1332 IU daily	No data	No data on HbA1c. Decrease of 21% in HOMA-IR but not significant	Insulin secretion and resistance improved
Sugden [33], 2008, UK	RCT, N = 87	8 weeks	100 000 IU vitamin D2	65 ± 10	HbA1c Change: 0.01 ± 0.60, p=.74. Change in HOMA-IS: −39.7 ± 79.3, p=.72	HOMA-IS improved in those with a 25-OHvitamin D increase of ≥11 nmol/l. No change in HbA1c
Witham [29], 2010, UK*	RCT, N = 61	8 weeks	Vitamin D3 100,000/200,000 IU single dose	65 ± 11	HbA1c 7.0 (1.6) vs. 7.1 (2.0 HOMA-IR 11.7 (12.7) vs. 13.5 (12.8)	HOMA-IR and HbA1c did not improve with either dose of vitamin D3
Talaei [19], 2013, Iran	Longitudinal study, N = 100	8 weeks	50,000 U vitamin D3 orally/week	54 + 11	No data on HbA1c HOMA-IR: 3.6 ± 3.2 vs. 2.9 ± 3.3	Significant improvement in HOMA-IR
Sabherwal [35], 2010, UK	Retrospective study, N = 52	3 months	400 IU vitamin D3	59 ± 8	HbA1c 8.9 ± 0.9% vs. 8.5 ± 0.8%, p <0.001 No data on HOMA parameters	HbA1c improved in both the vitamin D deficient and insufficient groups
Nikooyeh [22], 2011, Iran	RCT, N = 90	12 weeks	500 IU vitamin twice daily	51 ± 6	HbA1c −0.4 ± 1.2% (p < 0.001)	Significant improvement in HbA1c and HOMA-IR
					HOMA-IR 3.3 ± 1.8 vs. 2.7 ± 1.5	
Shab-bidar [23], 2011, Iran	RCT, N = 100	12 weeks	1,000 IU vitamin D3 daily	52.5 ± 7.4	HbA1c 8.7 ± 1.8 vs. 7.8 ± 1.3, p = 0.001 QUICKI: 0.29 ± 0.02 vs. 0.30 ± 0.02, p = 0.001	QUCIKI improved. HbA1c decreased but between-group changes non significant
Yiu [31], 2013, Hong Kong	RCT, N = 100	12 weeks	5000 IU vit. D3 daily	65 ± 8	HbA1c 7.35 vs. 7.20, p = 0.08 No data on HOMA	HbA1c similar between 2 groups
Bonakdaran [18], 2012, Saudi	Longitudinal study, N = 119	8 weeks	0.5 μg calcitriol daily	55 ± 11	HbA1c 8.4 + 1.8 vs. 7.1 ± 1.6, p = 0.01. No data on HOMA parameters	HbA1c improved
Heshmat [21], 2012, Iran	RCT, N = 42	3 months	Single IM 300,000 IU vitamin D3	56 ± 9	Percentage change in HbA1c: −0.01 ± 0.9	No positive effect
					HOMA-IR constant	
Eftekhari [37], 2011, Iran	RCT, N = 70	12 weeks	Calcitriol 0.25 μg daily	54 ± 9	HbA1c 7.1 ± 1.6 vs. 7.9 ± 2.1, p=.004	Attenuated the increase in glycaemia, and increased HOMA-B, but no effect on IR
					HOMA-IR 3.6 ± 2.5 vs. 4.8 ± 2.7	
					HOMA-B 3.4 ± 3.0 vs. 4.8 ± 3.8, p < 0.005	
Kota [36], 2011, India	RCT, n = 30	12 weeks	Oral cholecalciferol 60,000 units/week	38.4 ± 19.6	HbA1c 11.1 ± 1.3 to 7.7 ± 0.9 versus 10.3 ± 1.2 to 7.8 ± 1.1 (p > 0.1) in placebo. No data on HOMA parameters	HbA1c did not show significant improvement^1^
Soric [30], 2012, USA	RCT, N = 19	12 weeks	2000 IU vitamin D3 daily	54 ± 9	Change in A1c 0.4 ± 1.2, p = 0.16	Significant reduction in only in those with HbA1c >9.0%
					No data on HOMA parameters	
Kampmann [40], 2014, Denmark	RCT, n = 8 in each group	12 weeks	Cholecal ciferol^2^	62 ± 4.	ΔHbA1c 0.0004 ± 0.002, p = 0.07 vs. placebo	HbA1c and insulin sensitivity (hyperinsulinemia euglycemic clamp) did not change significantly
					Mild increase in insulin secretion	
Tabesh [39], 2014, Iran	RCT, N = 70	8 weeks	Cholecalciferol divided into 4 groups***	50.2 ± 6.6	HbA1c [−0.70 ± 0.19% (−8.0 ± 0.4 mmol/mol) p = 0.02] change from baseline. HOMA-IR (−0.46 ± 0.20, p = 0.001) change from baseline	Calcium–vitamin D co-supplementation resulted in improved HbA1c, HOMA-IR and QUICKI

IM: Intramuscular, RCT: Randomized controlled trial. *This study has provided data on both short term and long term changes in glycemic parameters.

**Studies with a follow up of ≤ 3 months were considered as short-term studies.

***(1) 50,000 U/week vitamin D + calcium placebo; (2) 1,000 mg/day calcium + vitamin D placebo (3) 50,000 U/week vitamin D + 1,000 mg/day calcium or (4) vitamin D placebo + calcium placebo.

^1^Newly diagnosed pulmonary TB cases with uncontrolled diabetes. ^2^Cholecalciferol 11200 IU daily for 2 weeks followed by 5600 IU daily.

Fourteen studies had assessed change in HbA1c over time after vitamin D supplementation. The study that had used calcitriol had the largest sample size (n = 119) and the results suggested a significant improvement in mean HBa1c after eight weeks from 8.4 to 7.1 [18]. Conversely the second study that had used calcitriol, found a paradoxical rise in HbA1c. But the sample size was smaller in this RCT (n = 35 in each group). However, authors noted that the use of calcitriol was associated with improvements in insulin secretion [37]. Of the remaining studies that had used cholecalciferol and assessed change in HbA1c over time, five studies found significant improvement in HbA1C at three months [22,23,30,35,39]. In the RCT conducted by Soric et al., patients with higher baseline HbA1c had a significantly greater reduction in HbA1C after 12 weeks [30]. Similarly, in the RCT conducted by Sugden et al., HOMA parameters significantly improved though HbA1c remained constant [33]. However, in the RCT that used a single intramuscular injection of vitamin D, HbA1c and HOMA-IR remained stable despite correction of low vitamin D status [21]. Similar lack of improvement in HbA1c and HOMA-IR were noted in the RCT conducted by Witham and colleagues. In this study, a large single dose of vitamin D3 was used [29]. Of particular note, one study that used hyperinsulinemic euglycemic clamp technique found that insulin sensitivity and HbA1c did not improve significantly [40]. In summary, ten short-term studies found improvement in HbA1c, HOMA-B and HOMA-IR though only four studies had analyzed all three outcomes [19,22,37]. QUICKI, an index of insulin sensitivity was shown to improve at 12 weeks in another RCT [23,39]. Certain parameters such as glycemic control and serum vitamin D levels may have affected the response in glycemic parameters. For instance, in one RCT, HbA1c improvement was noted only in subjects who had a baseline HbA1c greater than 9% [30]. Similarly, in the RCT conducted by Sugden et al., HOMA parameters significantly improved in patients who had a 25-OHvitamin D increase of 11 nmol/l or more [33].

### Clinical and biochemical characteristics of the long-term studies

There were nine studies identified as long-term studies. Six of them were RCTs. Data are shown in Table 2. Various studies had a sample size ranging from 22–204 and the duration of follow-up ranged from four to eighteen months. Eight studies had assessed the change in HbA1c over time after vitamin D supplementation. Most studies used oral cholecalciferol for supplementation. The dose of cholecalciferol ranged from 400–5700 IU daily to 40, 000 IU monthly. Calcitriol and vitamin D2 were not used in any of the long-term studies. A single dose intramuscular injection of vitamin D3 was used in two studies [29,38]. Only three studies had a follow up period beyond six months.

**Table 2:** Clinical and biochemical characteristics of the long-term studies**

**Author, year, country**	**Study design, N**	**Duration**	**Intervention**	**Age (Years)**	**Change in HbA1c and HOMA**	**comments**
Witham [29] 2010, UK*	RCT, N = 61	16 Weeks	Vitamin D3 100,000 or 200,000 IU single dose	65 ± 11	HbA1c 7.0 ± 1.6 vs. 6.9 ± 1.5	HOMA-IR and HbA1c did not improve
					HOMA-IR11.7 ± 12.7 vs. 15.9 ± 14.3	
Patel [34], 2010, USA	RCT, N = 24	4 Months	Vitamin D3 400 or 1200 IU	58 ± 3	HbA1c 6.7 ± 0.3 vs. 6.9 ± 0.2	QUICKI & HbA1c did not improve
					QUICKI 0.35 ± 0.01 vs. 0.35 ± 0.01^1^	
Jorde [32], 2009, Norway	RCT, N = 32	6 Months	Vitamin D3 (40,000 IU/week)	58 ± 10	HbA1c 8.0 ± 1.3 at baseline and changed by −0.2 ± 0.9 (p=.90 vs. change in the placebo group)	Insulin resistance and HbA1c did not change
					HOMA-IR 27.6 ± 34.3 at baseline and changed by 0.3 ± 23.5 (p=.58 vs. change in placebo group)	
Jehle [38], 2014, Switzerland	RCT, N = 55	6 Months	300,000 IM	67 ± 3	Significant intergroup difference in the ΔHbA1c (relative change + 2.9 ± 1.5% in the vitamin D group vs. +6.9 ± 2.1% in placebo group. HOMA-IR decreased by 13 ± 6% in the vitamin D group and increased by 10 ± 5% in the placebo group (p = 0.032)	Improved HOMA-IR and HbA1c
Strobel [15], 2014, Germany	RCT, N = 86	6 Months	Vigantol oil once a week (1904 IU)	61 (36–78)	Groups: 25 (OH) vitamin D >20 vs. ≤20 ng/ml.	No effects on metabolic parameters
					HbA1c 50 vs. 54 mmol/mol Hb, p = NS	
Huang [11], 2012, China	Longitudinal, N = 22	6 Months	Vitamin D3 800 IU/d	61 ± 10	HbA1c 7.1 ± 1.4 vs. 7.2 ± 1.4, p = 0.86	HbA1c remained stable
					No data on HOMA parameters	
Alam [16], 2014, UK	Retrospective audit, N = 204	8.0 ± 4.0 months	Vitamin D2 or vitamin D3***	61 ± 12	HbA1c 8.5 ± 1.7 vs. 8.0 ± 1.5 after treatment in ergocalciferol treated patients	HbA1c levels improved with ergocalciferol than cholecalciferol
					No data on HOMA parameters	
Breslavsky [17], 2013, Israel	RCT, N = 24	12 Months	Vitamin D3 1000 IU	67 ± 9	HbA1c 7.0 ± 1.1 vs. 7.3 ± 1.1, p = 0.212	No effect on glycemic parameters
					HOMA-IR 4.2 vs. 6.1, p=.243, HOMA-B 84.7 vs. 42.5, p = 0.184	
Al-Daghri [20], 2012, Saudi	Prospective longitudinal, N = 92	18 months	Vitamin D3 2000 IU	54 ± 10	No data on HbA1c	HOMA-B showed improvement until 18 months
					HOMA-B: 52 ± 9 vs. 97 ± 15, p = 0.002	

IM: Intramuscular, RCT: Randomized controlled trial. *This study has provided data on both short term and long term changes in glycemic parameters.

**Studies with a follow up of > 3 months were considered as long-term studies.

^1^QUICKI: An index of insulin sensitivity.

***Vitamin. D2 50,000 IU/d *10 d followed by 24,000 IU Vitamin D3/month OR Vitamin D3 40,000/d *10 d followed by 24,000/40,000 IU monthly.

Contrary to the results of the short-term studies, fewer long-term studies observed improvement in HbA1c [16,20,38]. Two studies that had a 4-month follow-up did not find any improvement in HOMA parameters or HbA1c [29,34]. Among the four studies with a follow up of six months, one study that used a higher supplementary dose of 40,000 IU per week observed that glycaemic control did not improve in subjects with normal serum 25-hydroxyvitamin D levels [32]. Conversely, another study that used a single high dose of intramuscular vitamin D, reported significant improvements in HOMA-IR after six months [38]. The study from Saudi Arabia had the longest follow up of 18 months and noted that HOMA-B improved over that period [20]. The beneficial effect was more pronounced in women than men. Interestingly the serum vitamin D levels were in the suboptimal range despite all subjects taking 2000 IU vitamin D daily [20]. HOMA parameters showed improvement only in two studies [20,38]. However HOMA measures remained stable in the remaining five studies in which this was assessed [15,17,29,32,34].

### Quality assessment

According to the Jadad scale, seven RCTs (11/17) included in this systematic review were of relatively high quality (Jadad score ≥3) and details are shown in Table 3. The quality assessment of the longitudinal cohort studies is presented in Table 4.

**Table 3:** **Quality assessment of eligible randomized controlled studies as assessed by the Jadad scale**

	**Was the study described asrandomized?**	**Was the method used to generate the sequence of randomization described and appropriate?**	**Was the study described as double blind?**	**Was the method of double blinding described and appropriate?**	**Was there a description of withdrawals and dropouts?**	**JADAD score**
Short term studies*						
Parekh [28]	1	1	1	0	1	4
Sugden [33]	1	1	1	0	1	4
Witham** [29]	1	1	1	1	1	5
Eftekhari [37]	1	0	1	0	0	2
Soric [30]	1	0	0	0	0	1
Heshmat [21]	1	0	1	0	0	2
Yiu [31]	1	1	1	1	1	5
Nikooyeh [22]	1	0	1	0	1	3
Shab-bidar [23]	1	0	1	0	0	2
Kota [36]	1	0	1	0	0	2
Tabesh [39]	1	1	1	’1	1	5
Kampmann [40]	1	1	1	1	0	4
Long term studies*						
Witham** [29]	1	1	1	1	1	5
Patel [34]	1	0	0	0	1	2
Jorde [32]	1	0	0	0	1	2
Jehle [38]	1	1	1	1	0	4
Strobel [15]	1	0	1	1	0	3
Breslavsky [17]	1	0	1	0	1	3

*Studies with a follow up of ≤ 3 months were considered as short-term studies. Studies with a follow up of > 3 months were considered as long-term studies.

**This study has provided data on both short term and long term changes in glycemic parameters.

**Table 4:** **Quality assessment of eligible longitudinal cohort studies as assessed by the Newcastle–Ottawa quality assessment scale**

	Short-term studies**				Long term-studies**		
**Selection**	**Sabherwal [** 35 **]**	**Borissova [** 27 **]**	**Talaei [** 19 **]**	**Bonakdaran [** 18 **]**	**Alam [** 16 **]**	**Huang [** 11 **]**	**Al-Daghri [** 20 **]**
Representativeness of the exposed cohort	*	*	*	*	*	*	*
Selection of the non- exposed cohort						*	*
Ascertainment of exposure	*	*	*	*	*	*	*
Demonstration that outcome was not present at start of study	*	*	*	*	*	*	*
**Comparability**							
Comparability of cohorts						*	
**Outcome**							
Assessment of outcome	*	*	*	*	*	*	*
Was follow-up long enough	*	*	*	*	*	*	*
Adequacy of follow up of cohorts	*	*	*	*	*	*	*

A study can be awarded a maximum of one star for each numbered item within the selection and outcome categories. A maximum of two stars can be given for comparability.

**Studies with a follow up of ≤ 3 months were considered as short-term studies. Studies with a follow up of > 3 months were considered as long-term studies.

## Discussion

This systematic review summarizes the most recent advances in our knowledge of evidence on how vitamin D supplementation affects glycemic parameters in patients with type 2 diabetes. There have not been many systematic reviews or meta-analyses on this topic [14,42,43]. Past narrative reviews have primarily focused on data obtained from preventive trials and observational studies [44]. We found 24 eligible studies out of which 17 were RCTs. Results of the various short-term studies included in this systematic review suggested that vitamin D supplementation had a positive impact on glycemic control and metabolic parameters such as insulin resistance and beta cell dysfunction. However, the evidence is weak due to the low methodological quality of the studies. Further, we found that there was no significant effect on HbA1c, beta cell function and insulin resistance based on the results of the long term studies that had an intervention period beyond three months. Only three long-term studies demonstrated a favorable profile but two of them were of inferior quality when compared to the remaining RCTs that demonstrated a lack of significant effect on glycemic parameters [16,20]. We also observed a lot of heterogeneity in the methodology of the studies (short term trials versus long term studies), inclusion criteria, supplementation of vitamin D (oral dose versus. intramuscular, ergocalciferol versus calcitriol or cholecalciferol) and the duration of follow up. Clearly there is a need for properly designed good quality RCTs with long term follow up to assess the potential beneficial effect of vitamin D supplementation in glycemic control, beta cell function and insulin resistance.

Our systematic review updates the findings of the previous meta-analyses. Our conclusions are similar to that of a recent meta-analysis [39]. But our review is more selective and detailed since we had focused specifically on intervention studies in patients with type 2 diabetes [39]. The meta-analysis by Seida et al. was different from our review in many ways [42]. Their meta-analysis may have missed out on some important studies due to the exclusion of non-RCT longitudinal studies [16,18-20,35]. They had also excluded studies in which synthetic vitamin D preparations or vitamin D2 were used. All the RCTs involved in this meta-analysis had sample sizes less than 50. In addition, some trials included in this meta-analysis had not studied vitamin D related glycemic outcomes as their primary analysis [23,36]. Our systematic review included 24 studies including 17 RCTs where as the meta-analysis by Seida et al. assessed data from 15 RCTs and the sample size for the pooled analysis was much smaller [42]. Nevertheless our conclusions are similar and further strengthen their conclusions that vitaminD3 supplementation might not decrease insulin resistance and hyperglycemia in patients with established type 2 diabetes. Similarly, another recent meta-analysis published in 2012 concluded that there was insufficient evidence to support a beneficial role of vitamin D on hyperglycaemia or insulin resistance [14]. However, this meta-analysis was conducted by pooling data from patients with normal glucose tolerance, impaired glucose tolerance and type 2 diabetes [29,33,45-47]. Also the literature search was restricted to March 2011 and hence they could only capture very limited number of studies. Many RCTs have been conducted since then and hence our systematic review contains more recent information. In a meta-analysis involving 328 patients and 6 RCTs, vitamin D supplementation was shown to improve HbA1c but failed to show any improvement in other parameters such as fasting blood glucose, quantitative insulin sensitivity check index (QUICKI), HOMA-B, and HOMA-IR [43].

Several possible explanations exist for the lack of beneficial effect of vitamin D on metabolic outcomes related to glucose. Factors related to vitamin D and diabetes may be attributed. First factor linked to vitamin D is related to its dosing. Sub optimal dosing of vitamin D may be one potential reason as not all studies documented correction of low vitamin D and high PTH. Some studies in the past have reported that the positive effect of vitamin D on beta-cell function and glucose tolerance is partly be due to correction of hypocalcaemia and secondary hyperparathyroidism [48]. The dose of vitamin D used may not have been adequate; most studies used daily doses of less than 2000 IU and daily doses up to 5000 IU may be essential to raise serum 25 (OH) vitamin D levels above the 75-nmol/L level. The appropriate dose of vitamin D that can achieve non-skeletal benefits still remains unclear. As observed in some studies, supraphysiological dosing of vitamin D may have been harmful [32]. Third, there could be differences between vitamin D2 and D3. For example, one study in this review observed that favorable change in HbA1c occurred only in patients treated with ergocalciferol during the initial course of the study [16]. Forth, baseline vitamin D status is a potential confounder on glycemic status and not many studies analyzed the effect of vitamin D supplementation on patients with vitamin D deficiency at baseline. Fifth, genetic factors related to vitamin D metabolism might play a role. It is likely that some ethnic groups might have a lower sensitivity to the effects of vitamin D and PTH. Individual variability may also be partly explained by vitamin D receptor polymorphisms. Moreover, the lack of significant association might have occurred due to the fact that the studies might have been underpowered with small sample size, short duration of intervention, lack of appropriate control groups and post hoc heterogeneity in the type and dose of formulations of vitamin D. Diabetes related reasons likely responsible for not finding a beneficial effect with vitamin D treatment include degree of hyperglycemia and duration of diabetes. Selective inclusion of patients with higher baseline glucose or HbA1c values may have been associated with greater improvements with vitamin D supplementation. In addition, the included subjects in some studies were treated with metformin and/or insulin, which might have masked the positive effects of vitamin D [23,32]. Next, only one study had used the gold-standard method of hyperglycemic clamp (for insulin secretion or sensitivity), but no significant association was noted [40].

The strengths and limitations of this systematic review need mention. Although we performed a comprehensive search of electronic literature some studies could exist that have not been included. Other limitations of our study are based on the quality and methodological flaws of the included studies as well as the lack of availability of sufficient information from eligible studies. First, detailed information about confounding factors such as ethnicity, physical activity, baseline HbA1c, body mass index, obesity, sun exposure, seasonal changes in vitamin D, dietary vitamin D intake, calcium intake, vitamin D receptor polymorphisms, baseline vitamin D status, and compliance with vitamin D supplements that could influence the response of vitamin D was not available in all of the included studies [49]. Data was also lacking regarding the compliance with supplements and possible gender differences in metabolic effects. Second, almost all studies assessed insulin secretion and resistance based on HOMA related parameters that are not as accurate as the glucose clamp techniques. It can be argued that clamp techniques are invasive to be conducted in large-scale studies. But there are more reliable dynamic tests now being used to assess insulin sensitivity and beta cell function. Furthermore, most trials concentrated only on short-term or intermediate effects, such as glycemic status and insulin resistance. Long-term trials investigating the occurrence of micro and macrovascular complications were seldom conducted and such trials may bring in a new dimension to the field. Some studies failed to meet the endpoints for improved insulin sensitivity and beta cell function though confirmed improvement in HbA1c [23,37]. Lack of statistical analysis limits our ability to make a single conclusion from different studies. Finally, the included studies were conducted mostly in Caucasians making the results less generalizable to other racial groups. Our systematic review has several strengths. This review is based on an up to date literature search represents the most extensive review on this topic so far. The eligible studies were either RCTs or longitudinal studies. Most studies had prospective designs, adding more strength to our results. The data were extracted from well-reviewed studies using a comprehensive literature search. We relied on duplicate independent judgment and there was sufficient inter-observer agreement. The extensive review shows that the evidence is not of good quality, mainly because of the many methodological limitations of the included studies. Our systematic review underscores the need for future studies given that both vitamin D deficiency and diabetes are conditions with huge public health concern worldwide.

## Conclusions

Based on our critical review, we conclude that currently available evidence based on randomized controlled trials and longitudinal studies suggest that vitamin D supplementation might not improve hyperglycemia, beta cell secretion or insulin sensitivity in patients with established type 2 diabetes. This shows that the pathogenetic and therapeutic role of vitamin D in glucose metabolism is still unclear. Experimental studies as well as large scale RCTs with good study design, optimal vitamin D supplementation and long-term follow up are needed on this topic.

## Abbreviations

RCTS: Randomized controlled trials (RCTs)
HOMA-B: Homeostasis model assessment index of beta cell function
HOMA-IR: Homeostasis model assessment index of insulin resistance

## References

[1] Chan JC, Malik V, Jia W, Kadowaki T, Yajnik CS, Yoon KH (2009). Diabetes in Asia: epidemiology, risk factors, and pathophysiology. JAMA.

[2] Hu FB (2011). Globalization of diabetes: the role of diet, lifestyle, and genes. Diabetes Care.

[3] Group UKPDS (1998). Intensive blood-glucose control with sulphonylureas or insulin compared with conventional treatment and risk of complications in patients with type 2 diabetes (UKPDS 33). Lancet.

[4] DCCT Research Group (1993). The effect of intensive treatment of diabetes on the development and progression of long-term complications insulin-dependent diabetes mellitus. N Engl J Med.

[5] Wallace TM, Matthews DR (2000). Poor glycaemic control in type 2 diabetes: a conspiracy of disease, suboptimal therapy and attitude. QJM.

[6] Montane J, Cadavez L, Novials A (2014). Stress and the inflammatory process: a major cause of pancreatic cell death in type 2 diabetes. Diabetes Metab Syndr Obes.

[7] Faria HT, Santos MA, Arrelias CC, Rodrigues FF, Gonela JT, Teixeira CR (2014). Adherence to diabetes mellitus treatments in family health strategy units. Rev Esc Enferm USP.

[8] Personne V, Partouche H, Souberbielle JC (2013). Vitamin D insufficiency and deficiency: epidemiology, measurement, prevention and treatment. Presse Med.

[9] Garland CF, Kim JJ, Mohr SB, Gorham ED, Grant WB, Giovannucci EL (2014). Meta-analysis of All-Cause Mortality According to Serum 25-Hydroxyvitamin D. Am J Public Health.

[10] Mezza T, Muscogiuri G, Sorice GP, Prioletta A, Salomone E, Pontecorvi A (2012). Vitamin D deficiency: a new risk factor for type 2 diabetes. Ann Nutr Metab.

[11] Huang Y, Yu H, Lu J, Guo K, Zhang L, Bao Y (2012). Oral supplementation with cholecalciferol 800 IU ameliorates albuminuria in Chinese type 2 diabetic patients with nephropathy. PLoS One.

[12] Mitri J, Muraru MD, Pittas AG (2011). Vitamin D and type 2 diabetes: a systematic review. Eur J Clin Nutr.

[13] Song Y, Wang L, Pittas AG, Del Gobbo LC, Zhang C, Manson JE (2013). Blood 25-hydroxy vitamin D levels and incident type 2 diabetes: a meta-analysis of prospective studies. Diabetes Care.

[14] George PS, Pearson ER, Witham MD (2012). Effect of vitamin D supplementation on glycaemic control and insulin resistance: a systematic review and meta-analysis. Diabet Med.

[15] Strobel F, Reusch J, Penna-Martinez M, Ramos-Lopez E, Klahold E, Klepzig C (2014). Effect of a randomised controlled vitamin D trial on insulin resistance and glucose metabolism in patients with type 2 diabetes mellitus. Horm Metab Res.

[16] Alam U, Chan AW, Buazon A, Van Zeller C, Berry JL, Jugdey RS (2014). Differential effects of different vitamin D replacement strategies in patients with diabetes. J Diabet Complicat.

[17] Breslavsky A, Frand J, Matas Z, Boaz M, Barnea Z, Shargorodsky M (2013). Effect of high doses of vitamin D on arterial properties, adiponectin, leptin and glucose homeostasis in type 2 diabetic patients. Clin Nutr.

[18] Bonakdaran S, Hami M, Hatefi A (2012). The effects of calcitriol on albuminuria in patients with type-2 diabetes mellitus. Saudi J Kidney Dis Transpl.

[19] Talaei A, Mohamadi M, Adgi Z (2013). The effect of vitamin D on insulin resistance in patients with type 2 diabetes. Diabetol Metab Syndr.

[20] Al-Daghri NM, Alkharfy KM, Al-Othman A, El-Kholie E, Moharram O, Alokail MS (2012). Vitamin D supplementation as an adjuvant therapy for patients with T2DM: an 18-month prospective interventional study. Cardiovasc Diabetol.

[21] Heshmat R, Tabatabaei-Malazy O, Abbaszadeh-Ahranjani S, Shahbazi S, Khooshehchin G, Bandarian F (2012). Effect of vitamin D on insulin resistance and anthropometric parameters in Type 2 diabetes; a randomized double-blind clinical trial. Daru.

[22] Nikooyeh B, Neyestani TR, Farvid M, Alavi-Majd H, Houshiarrad A, Kalayi A (2011). Daily consumption of vitamin D or vitamin D calcium-fortified yogurt drink improved glycemic control in patients with type 2 diabetes: a randomized clinical trial. AmJ Clin Nutr.

[23] Shab-Bidar S, Neyestani TR, Djazayery A, Eshraghian MR, Houshiarrad A, Gharavi A (2011). Regular consumption of vitamin D-fortified yogurt drink (Doogh) improved endothelial biomarkers in subjects with type 2 diabetes: a randomized double-blind clinical trial. BMC Med.

[24] Moher D, Liberati A, Tetzlaff J, Altman DG (2009). Preferred reporting items for systematic reviews and meta-analyses: the PRISMA statement. BMJ.

[25] Wells GS, B O’Connell, D Peterson, J Welch, V Losos, M Tugwell. P. The Newcastle-Ottawa Scale (NOS) for assessing the quality of nonrandomized studies in meta-analysis.2011. Access date: July 2014. www.ohri.ca/programs/clinical_epidemiology/oxford.htm.

[26] Jadad AR, Moore RA, Carroll D, Jenkinson C, Reynolds DJM, Gavaghan DJ (1996). Assessing the quality of reports of randomized clinical trials: Is blinding necessary?. Control Clin Trials.

[27] Borissova AM, Tankova T, Kirilov G, Dakovska L, Kovacheva R (2003). The effect of vitamin D3 on insulin secretion and peripheral insulin sensitivity in type 2 diabetic patients. Int J Clin Pract.

[28] Parekh D, Sarathi V, Shivane VK, Bandgar TR, Menon PS, Shah NS (2010). Pilot study to evaluate the effect of short-term improvement in vitamin D status on glucose tolerance in patients with type 2 diabetes mellitus. Endocr Pract.

[29] Witham MD, Dove FJ, Dryburgh M, Morris AD, Struthers AD (2010). The effect of different doses of vitamin D3 on markers of vascular health in patients with type 2 diabetes—a randomised controlled trial. Diabetologia.

[30] Soric MM, Renner ET, Smith SR (2012). Effect of daily vitamin D supplementation on HbA1c in patients with uncontrolled type 2 diabetes mellitus: a pilot study. J Diab.

[31] Yiu YF, Yiu KH, Siu CW, Chan YH, Li SW, Wong LY (2013). Randomized controlled trial of vitamin D supplement on endothelial function in patients with type 2 diabetes. Atherosclerosis.

[32] Jorde R, Figenschau Y (2009). Supplementation with cholecalciferol does not improve glycaemic control in diabetic subjects with normal serum 25-hydroxyvitamin D levels. Eur J Nutr.

[33] Sugden JA, Davies JI, Witham MD, Morris AD, Struthers AD (2008). Vitamin D improves endothelial function in patients with Type 2 diabetes mellitus and low vitamin D levels. Diabet Med.

[34] Patel P, Poretsky L, Liao E (2010). Lack of effect of subtherapeutic vitamin D treatment on glycemic and lipid parameters in Type 2 diabetes: A pilot prospective randomized trial. J Diab.

[35] Sabherwal S, Bravis V, Devendra D (2010). Effect of oral vitamin D and calcium replacement on glycaemic control in South Asian patients with type 2 diabetes. Int J Clin Pract.

[36] Kota SK, Jammula S, Kota SK, Tripathy PR, Panda S, Modi KD (2011). Effect of vitamin supplementation in type 2 diabetes patients with pulmonary tuberculosis. Diab Metab Syndr.

[37] Eftekhari MH, Akbarzadeh M, Dabbaghmanesh MH, Hasanzadeh J (2011). Impact of treatment with oral calcitriol on glucose indices in type 2 diabetes mellitus patients. Asia Pac J Clin Nutr.

[38] Jehle S, Lardi A, Felix B, Hulter HN, Stettler C, Krapf R (2014). Effect of large doses of parenteral vitamin D on glycaemic control and calcium/phosphate metabolism in patients with stable type 2 diabetes mellitus: a randomised, placebo-controlled, prospective pilot study. Swiss Med Wkly.

[39] Tabesh M, Azadbakht L, Faghihimani E, Tabesh M, Esmaillzadeh A (2014). Effects of calcium-vitamin D co-supplementation on metabolic profiles in vitamin D insufficient people with type 2 diabetes: a randomised controlled clinical trial. Diabetologia.

[40] Kampmann U, Mosekilde L, Juhl C, Moller N, Christensen B, Rejnmark L et al. Effects of 12weeks high dose vitamin D3 treatment on insulin sensitivity, beta cell function, and metabolic markers in patients with type 2 diabetes and vitamin D insufficiency- a double-blind, randomized, placebo-controlled trial. Metabolism. 2014. doi:10.1016/j.metabol.2014.06.008 10.1016/j.metabol.2014.06.008 25044176

[41] Krul-Poel YH, Wijland H, Stam F, Ten Boekel E, Lips P, Simsek S (2014). Studyprotocol: a randomised placebo-controlled clinical trial to study the effect of vitamin D supplementation on glycaemic control in type 2 Diabetes Mellitus SUNNY trial. BMC Endocr Disord.

[42] Seida JC, Mitri J, Colmers IN, Majumdar SR, Davidson MB, Edwards AL (2014). Effect of Vitamin D (3) Supplementation on Improving Glucose Homeostasis and Preventing Diabetes: A Systematic Review and Meta-Analysis. J Clin Endocrinol Metab.

[43] Gao W, Chen DW, Liu GJ, Ran XW (2013). Efficacy and safety of vitamin D for type 2 diabetes mellitus: a systematic review. Zhonghua Yi Xue Za Zhi.

[44] Khan H, Kunutsor S, Franco OH, Chowdhury R (2013). Vitamin D, type 2 diabetes and other metabolic outcomes: a systematic review and meta-analysis of prospective studies. Proc Nutr Soc.

[45] Jorde R, Strand Hutchinson M, Kjærgaard M, Sneve M, Grimnes G (2013). Supplementation with High Doses of Vitamin D to Subjects without Vitamin D Deficiency May Have Negative Effects: Pooled Data from Four Intervention Trials in Tromsø. ISRN Endocrinol.

[46] Ljunghall S, Lind L, Lithell H, Skarfors E, Selinus I, Sorensen OH (1987). Treatment with 1α-hydroxycholecalciferol in middle-aged men with impaired glucose-tolerance- a prospective randomized double-blind-study. Acta Med Scand.

[47] Pittas AG, Lau J, Hu FB, Dawson-Hughes B (2007). The role of vitamin D and calcium in type 2 diabetes. A systematic review and meta-analysis. J Clin Endocrinol Metab.

[48] Kumar S, Davies M, Zakaria Y, Mawer EB, Gordon C, Olukoga AO (1994). Improvement in glucose tolerance and beta-cell function in a patient with vitamin D deficiency during treatment with vitamin D. Postgrad Med J.

[49] Neyestani TR, Djazayery A, Shab-Bidar S, Eshraghian MR, Kalayi A, Shariátzadeh N (2013). Vitamin D Receptor Fok-I polymorphism modulates diabetic host response to vitamin D intake: need for a nutrigenetic approach. Diabetes Care.

